# A Fluorescent Reporter-Based Evaluation Assay for Antibacterial Components Against *Xanthomonas citri* subsp. *citri*

**DOI:** 10.3389/fmicb.2022.864963

**Published:** 2022-05-04

**Authors:** Yunfei Long, Ruifang Luo, Zhou Xu, Shuyuan Cheng, Ling Li, Haijie Ma, Minli Bao, Min Li, Zhigang Ouyang, Nian Wang, Shuo Duan

**Affiliations:** ^1^China-USA Citrus Huanglongbing Joint Laboratory, National Navel Orange Engineering Research Center, Gannan Normal University, Ganzhou, China; ^2^College of Agricultural and Food Sciences, Zhejiang A&F University, Hangzhou, China; ^3^Department of Microbiology and Cell Science, Citrus Research and Education Center, University of Florida, Lake Alfred, FL, United States

**Keywords:** antagonistic, *Xanthomonas citri*, evaluation and screening, citrus canker, reporter-based

## Abstract

*Xanthomonas citri* subsp. *citri* (*Xcc*) is the agent of citrus bacterial canker (CBC) disease, which has significantly reduced citrus quantity and quality in many producing areas worldwide. Copper-based bactericides are the primary products for CBC control and management, but the problems derived from copper-resistant and environmental contamination have become issues of anxiety. Thus, there is a need to find alternative antibacterial products instead of relying on a single type of agent. This study developed a method to evaluate the inhibition of antibacterial agents using the fluorescence-labeled recombinant *Xcc* strain (*Xcc-eYFP*). The optimization of timelines and parameters for the evaluation of antibacterial agents involved the use of a Spark™ multimode microplate reader. This evaluation and screening method can be applied to bactericides, cocktail-mixture formulations, antagonistic bacteria, and derived metabolites. The results showed that the minimum inhibitory concentration (MIC) of commercial bactericides determined by fluorescence agrees with the MIC values determined by the conventional method. A screened cocktail-mixture bactericide presents more activity than the individual agents during the protective effects. Notably, this method has been further developed in the screening of *Xcc*-antagonistic bacterial strains. In summary, we provide a validated strategy for screening and evaluation of different antibacterial components for inhibition against *Xcc* for CBC control and management.

## Introduction

Citrus is one of the essential fruit tree crops worldwide concerning total production and economic value (Sangiorgio et al., 2020). Citrus bacterial canker (CBC) disease is one of the most severe bacterial diseases harming the citrus industry (Li and Wang, 2011a; Rabbee et al., 2019). The causal agent is composed of strains that have been designated as *Xcc*A, *Xcc*A^w^, *Xcc*A*, *Xcc*B, and *Xcc*C according to host range and ability to elicit a hypersensitive response (HR) in different citrus varieties (Leduc et al., 2011; Ference et al., 2018). The *Xanthomonas citri* subsp. *citri* Asiatic strain *Xcc*A was distributed in many citrus-producing countries with high pathogenicity and a broad host range. *Xcc*A invades citrus leaves, stems, and fruit *via* natural openings such as stomata and wounds, further reducing citrus quality and productivity by causing severe defoliation, blemished fruit, and premature fruit drop. Theoretically, *Xcc*A causes hypertrophy and hyperplasia symptoms by secreting PthA4 and functional homologs, which are transcriptional activator-like effectors, *via* the type III secretion system (T3SS) into the nucleus of plant cells to induce the expression of the canker susceptibility gene *CsLOB*1 (Yan et al., 2012; Hu et al., 2014, 2016; Duan et al., 2018). In addition to T3SS and T3SS effectors, virulence factors involved in biofilm (Rigano et al., 2007; Li and Wang, 2011a), xanthan gum (Li and Wang, 2012), lipopolysaccharides (Li and Wang, 2011b; Yan and Wang, 2011; Petrocelli et al., 2012; Yan et al., 2012), and epiphytic fitness (Rigano et al., 2007) also play essential roles in the infection of *Xcc*.

In endemic canker regions, the primary control strategy is the application of copper-based bactericides to prevent *Xcc* infection (Narciso et al., 2012; Scapin et al., 2015; Gochez et al., 2018; Behlau et al., 2021b). Copper-based bactericides, in the presence of water and low pH, release copper ions to bind to proteins of pathogenic bacteria, leading to protein misfunction, proteins and nucleic acid damage, and ultimately the death of pathogens (Behlau et al., 2011). Even though the application of copper is not practical for protecting young, susceptible citrus foliage against *Xcc* infection, copper sprays still showed the highest contribution to canker control by reducing disease incidence and crop losses (Behlau et al., 2017; Heydarpanah et al., 2020; Machado et al., 2021). In contrast, the extensive use of copper-based bactericides has led to soil and water contamination that directly harms humans and surrounding ecosystems (Zhang et al., 2003). Copper accumulation has been reported to reduce microbial biomass and diversity in copper-affected soils (Zhou et al., 2011). Moreover, long-term use of copper-based bactericides has led to the development of copper-resistant strains in many phytopathogenic bacteria, including *Xcc*, resulting in a reduction of disease control (Behlau et al., 2011, 2012a). Even though the copper-resistant *Xcc* strain was only reported in Argentina and Reunion Island (Behlau et al., 2012b; Richard et al., 2017), it could appear under reliance on the extensive use of copper-based bactericides for disease management.

Disease-resistant crop varieties are efficient and environmentally friendly approaches to disease management (de Carvalho et al., 2015; Fu et al., 2020). Citrus-producing regions without CBC rely mainly on quarantine measures to keep the groves free of *Xcc*. Windbreaks also have a positive control effect on CBC by reducing wounds (Behlau et al., 2021a). Diverse and integrated disease control can reduce the risk of the application of copper-based bactericides during CBC control and management, but chemical control is still the primary method for preventing *Xcc* worldwide. Some substitutes for copper-based bactericides have been applied to CBC control. For example, imidacloprid and systemic acquired resistance-inducing compounds applied to soil (Graham and Myers, 2011); exogenous application of nicotinamide adenine dinucleotide by soil drenches or leaf infiltration (Alferez et al., 2018); foliar spraying of nanoformulated zinc oxide (Graham and Myers, 2016); and biofilm inhibitors (Li and Wang, 2014) and the root application of rhizobacteria (Riera et al., 2018) have positive control effects on CBC.

The application of new or mixed antibacterial components is a substitute strategy for copper-based bactericides, which can reduce the side effects of extensive use of copper-based bactericides separately (Ibrahim et al., 2017). Field protection revealed that the combination of bactericides presents a better performance effect than a single agent. However, the combination ratio of bactericides derived a significant number of combinations. Picking out the well-performance ratio from combinations requires an efficient approach instead of conventional methods because these are usually time-consuming, laborious, and less accurate. Minimum inhibitory concentration (MIC) is conventionally based on the absorption value and plate colony count, which is time-consuming and laborious. The inhibition zone method is commonly used for antagonism to evaluate bactericides against bacteria, based on the scope of the inhibition zone on the plate and the parameter MIC, which is affected by the nurturing plate and environment. Here, we firstly developed a rapid evaluation method based on eYFP labeling of *Xcc* strains. This study evaluated the inhibition rates of commercial bactericides, corresponding mixed formulations, *Xcc*-antagonistic bacterial strains, and derived products using a reporter-based assay. Subsequently, this method was evaluated for the indoor inhibition activity and field protective effect of bactericides.

## Materials and Methods

### Bacterial Strains, Plants, and Growth Conditions

*Xcc* strains were isolated from the Jiangxi province, China. All bacterial strains were maintained in 15% glycerol and preserved in a freezer at –80°C. *Escherichia coli* cells were cultured in the Luria–Bertani (LB) medium (tryptone 10 g/L, yeast extract 5 g/L, and NaCl 10 g/L) at 37°C. *Xcc* strains were recovered and cultured on nutrient broth (NB) medium (beef extract 3 g/L and peptone 5 g/L) and on nutrient agar (NA) medium (beef extract 3 g/L, peptone 5 g/L, and agar 12 g/L) plates at 28°C. When required, growth media were supplemented with gentamicin (20 μg/ml) and kanamycin (50 μg/ml).

Hamlin sweet orange [*Citrus sinensis* (L.) Osbeck] was grown in a greenhouse with a 16-h light and 8-h dark photoperiod, a 28/26°C temperature cycle, and 80% humidity. Fully expanded young leaves of approximately 1-month old were used.

### Construction of eYFP Plasmid

The plasmid was constructed as described in previous research (Duan et al., 2021). The plasmid DNA concentration was measured with the NanoDrop^®^ ND-1000 (NanoDrop, United States). Briefly, the broad-host-range vector pBBR1-eYFP was constructed by cloning the *eYFP* coding region into pBBR1-MCS5. The construct for pBBR1-eYFP was introduced into an *E. coli* DH5α competent cell and then selected on LB plates containing 20 μg/ml of gentamycin. Plasmid DNA extracted from *E. coli* transformants was reintroduced into *Xcc* competent cells using electroporation of the Gene Pulser Xcell system (Bio-Rad, United States) under the following conditions: 1 mm cuvette, voltage of 2,400 V; capacitance of 25 μF. The reconstructed *Xcc* strains were spread on NA plates containing 20 μg/ml of gentamycin. Polymerase chain reaction (PCR) and restricted enzymatic digestion were applied for the confirmation of plasmids. Meanwhile, the *Xcc-eYFP* strain can represent the fluorescent signal (excitation/emission at 514/527 nm) at 24-h post-transformation. The bacterial cell, clones, and suspension of *Xcc-eYFP* can be observed under a handheld lamp (#LUYOR-3260CY, LUYOR, China) and a fluorescent microscope (Leica, Germany) with corresponding filters.

### *Xanthomonas citri* subsp. *citri* Strain Susceptibility to Solvents

The susceptibility of *Xcc-eYFP* strains to solvents were examined under laboratory conditions: 10 mM MgCl_2_, liquid medium NB, dimethyl sulfoxide (DMSO), dimethylformamide (DMF), and acetone. A logarithmic culture of *Xcc-eYFP* was diluted into OD_600_ of 0.3 [about 5 × 10^8^ colony-forming units/ml (CFU/ml)] and suspended in a range of solvent concentration from 0 to 100% (20% of the interval). Plates were incubated at room temperature, and the luminescence of *Xcc-eYFP* was read at 0, 6, 12, 24, and 48 h using a Spark™ multimode microplate reader (TECAN, Switzerland) for fluorescent signal detection. Susceptibility to solvents was calculated as percentage inhibition using the formula [(negative control signal – sample signal)/negative control signal × 100].

### Bactericide Treatments and Pathogenicity Test

Commercial bactericides: 33% kasugamycin xine-copper (SC), 1.2% xinjunan acetate (AS), 30% copper oxychloride (SC), and 20% resin acid copper (EW) were diluted to the corresponding concentrations and mixed in a series of synergistic ratios with the corresponding solvent (Supplementary Tables 1, 2). The control effect of four individual bactericides (2 mg/ml) and three mixture formulations of bactericides were investigated. Briefly, Hamlin sweet orange leaves (2–3 weeks after leaf emergence) of 3–4-year-old citrus plants were punctured with five pins at six inoculation sites. Then, we sprayed these leaves with a bactericide or mock treatment. Twelve hours later, *Xcc-eYFP* suspensions were sprayed at the concentration of 5 × 10^8^ CFU/ml on the same inoculation sites. The treated leaves were cultured at a greenhouse at 28°C, with 80% relative humidity and a 16/8 h light/dark photocycle. Eight days post-spray treatment, the symptoms of the inoculated leaves were observed and photographed with a digital Canon-EOS 200D camera (Canon, Japan). The measurement process of the bacterial *Xcc-eYFP* population is described as follows: a 6-mm-diameter leaf disc, containing only a single puncture site, was excised from inoculated leaves 8 days after inoculation using a punch. Three biological repeats were collected and homogenized in 0.2 ml of double-distilled water (ddH_2_O) in 1.5-ml Eppendorf tubes. The homogenized solution was centrifuged at 1,000 rpm for 5 s to precipitate the debris. The upper phase with *Xcc-eYFP* was collected, and 10 μl of the solution was loaded into a Helber counting chamber (Auvon, United Kingdom) to be observed under a Leica DM3000 fluorescence microscope (Leica, Germany). The number of fluorescence spots in the square of the Helber counting chamber was counted. This procedure was repeated to collect the average for bacterial population calculation and then calculated following the formula in the Helber counting chamber instructions. Each experiment was repeated three times.

### Reporter-Based Determination of Minimum Inhibitory Concentrations

The MIC, defined as the lowest drug concentration at which more than 99% of bacterial growth is inhibited, was calculated from the fitted curve compared to the untreated control. Two methods were tested in this study. The first conventional method: *Xcc* and *Xcc-eYFP* strain, was grown in NB at 28°C with shaking at 200 rpm for 8 h. The cultures were standardized to an OD_600_ of 0.03 (about 5 × 10^6^CFU/ml) in NB and then aliquoted 190 μl into wells of a 96-well plate. Initial test concentrations of the compounds were diluted (1:20) in culture (10 μl of the compound in 190 μl of culture) and incubated at 28°C. Cultures were monitored at 24 and 48 h at OD_600_, and the lowest concentration resulting in no growth after 48 h compared with control samples was defined as the MIC for *Xcc* or *Xcc-eYFP*. All determinations were conducted in eight replicate wells and repeated three times. The second fluorescent-based method: *Xcc* and *Xcc-eYFP* cultures were diluted from a logarithmic phase culture in NB liquid medium and added to the appropriate 96 wells microtiter plates at a final OD_600_ of 0.03 (about 5 × 10^6^CFU/ml). And, 190 μl were aliquoted into the wells of a 96-well plate. The initial test concentrations of the compounds were diluted (1:20) in the culture (10 μl of the compound in 190 μl of culture). The plates were incubated at 28°C, and luminescence and fluorescence were read at 0, 6, 12, 24, and 48 h. A Gompertz model was used to fit the data and generate dose–response curves using GraphPad Prism (GraphPad Software, United States).

### The Inhibition Zone Method

A certain amount of *Xcc-eYFP* suspension was poured into the NA medium that had been cooled to about 50°C, mixed evenly, poured into the 80-mm plate (about 80 ml/plate), and let it stand horizontally for use after solidification. The bactericides were diluted stepwise and 11 concentration gradients were prepared for testing. Holes were punched in the test plate with a sterilized steel pipe, small pieces of medium were carefully picked out to make round holes, 80 μl of bactericides were injected into the holes and incubated at 28°C for 48 h. A Vernier caliper was applied to measure the zone of inhibition around the specimens in centimeters. The inhibition activity of bactericide can be preliminarily determined according to the diameter of the inhibition zone. The inhibition ratios were calculated: Inhibition rate (%) = (*R*_*t*_-*R*_*o*_)/*R_*o*_* × 100%, where *R*_*t*_ is the average diameter of the treatment group, and *R*_*o*_ is the average diameter of the control group. The experiments were performed in triplicate.

### Plant Tested Bactericide Mixture Formulation Using the Outdoor Spray Method

The control effect of a selected 1:1 mixture formulation of bactericides was investigated using a greenhouse with 20-week-old potted citrus lemon [*Citrus limon* (L.) Burm. f.] plants. The bacterial inoculum was prepared and suspended in ddH_2_O, and the concentration was adjusted to approximately OD_600_ = 0.3 (5 × 10^8^CFU/ml). The treatment was performed using a spray method as previously described (Duan et al., 2021). Briefly, the abaxial surfaces of flush leaves (2–3 weeks after leaf emergence) and immature leaves were sprayed with the following treatments: *Xcc* and *Xcc-eYFP* strain combined with three 1:1 synergistic bactericides of 1.2% xinjunan acetate (AS) (0.5 mg/ml) mix 33% kasugamycin xine-copper (SC) (0.25 mg/ml), 1.2% xinjunan acetate (AS) (0.5 mg/ml) mix 20% resin acid copper (EW) (0.03 mg/ml), and 1.2% xinjunan acetate (AS) (0.5 mg/ml) mix 30% copper oxychloride (SC) (0.03 mg/ml). These mixed formulations were added to each treatment at a final concentration of 0.03% (vol/vol). *Xcc* strain and *Xcc-eYFP* strain combined with ddH_2_O were used as controls. At 28 days post-spray treatment, leaf symptoms were observed and photographed with a digital camera.

For bacterial population assays, the leaves of citrus lemon plants were inoculated as described above. Two randomly selected leaf discs were cut from three inoculated leaves with a cork borer (6 mm of diameter). The bacterial population of the *Xcc-eYFP* strain was measured by the method described above. For the *Xcc* strain bacterial population, the leaf discs were ground in 1 ml of ddH_2_O. Suspensions were serially diluted and plated on NA plates. After incubation at 28°C for 48 h, bacterial colonies were counted, and the number of CFU/cm^2^ of the leaf tissue was calculated. The assays were repeated three times independently.

### Evaluation of Reporter-Based Bactericides

*Xcc* and *Xcc-eYFP* strains were collected on the corresponding plate, rinsed with ddH_2_O three times, and finally diluted in 10 mM MgCl_2_ at a density (OD_600_ = 0.6). 96-well plates were designed for screening treatment with the corresponding control. Bactericides or mixture formulations were dissolved in the corresponding buffer with the designed synergistic ratios. Then, they were proportioned with related bacterial suspension (OD_600_ = 0.6) of *Xcc-eYFP*, *Xcc* and the mock treatment into 96-well plates (Black 96-well microplate, flat bottom, Falcon). A total of 200 μl of the solution, including bacterial suspension, bactericides, or mock treatment, was pipetted into the well and incubated at 28°C for 6 h. Next, for *Xcc* strain, the suspension from the wells was determined by plating 10 μl of 10-fold serial dilutions on the NA plate and counting the resulting colonies. To *Xcc-eYFP* strain, 10 μl of the suspension from the wells was diffused into a Helber counting chamber for fluorescent spot counting using a Leica DM3000 fluorescence microscope (Leica Microsystems, Wetzlar, Germany). The number of fluorescent spots in the square of the Helber counting chamber was counted. This procedure was repeated to determine the average for bacterial population calculation. The bacterial population was calculated following the formulas in the instructions provided by the manufacturers of the Helber counting chamber A30000 (Auvon, Tonbridge, United Kingdom).

### Calculation of Reporter-Based Inhibition Rate

Wild-type *Xcc* and *Xcc-eYFP* without bactericides were applied as a control and three replicates for each treatment postincubation at 28°C for 6 h. The fluorescence intensity at an excitation/emission wavelength of 485/535 nm was measured using the Spark™ Multimode Microplate reader. Inhibition rates were calculated as percentage inhibition using the formula [(negative control signal - sample signal)/negative control signal × 100].

### Evaluation of *Xanthomonas citri* subsp. *citri*-Antagonistic Bacterial Strains

Bacterial strains were cultured in the LB or NB medium. Bacteria stock 15% glycerol was streak cultured on NA plates at 28°C for 2 days and then re-cultured in the corresponding medium. The potential antagonistic metabolites of bacteria were induced in liquid LB/NB medium at 28°C and 150 rpm in a shaking culture machine. The supernatant of culture suspension was then collected by centrifugation at 4,000 rpm. The supernatant containing potential antibacterial compounds was processed following the experiment of antagonistic strain screening. About 10 μl of *Xcc-eYFP* bacterial suspension (OD_600_ = 0.6) was added to 1 ml of fresh LB/NB medium with gentamycin (20 mg/ml) in a sterile 2-ml Eppendorf tube. The above supernatant was mixed proportionately into the tube and then cultured on a 28°C incubation shaker at 200 rpm for 12 h. *Xcc-eYFP* was precipitated at 13,000 rpm for 5 min and then resuspended by 200 μl of MgCl_2_ (10 mM). The resuspended bacterial suspensions were loaded on designed 96-well plates with controls, which were further delivered into the Spark™ multimode microplate reader (TECAN, Switzerland) for fluorescent signal detection. The collected data will be processed into the visual quantification below.

### Extraction and Evaluation of Antibacterial Components

Metabolites were extracted according to the following steps: ethyl acetate was selected for the metabolite extraction process because of its low boiling point and moderate polarity. Seed liquid cultures (5 ml NB) were re-cultured to 200 ml of NB in 0.5-L Erlenmeyer flasks at 28°C with 180 rpm for 36 h until the OD_600_ reached 1. An equal volume of ethyl acetate was added to the bacterial cultures, and the flasks were sonicated for 5 min and maintained overnight with vigorous shaking. Then, the culture broth was centrifuged at 3,500 rpm for 15 min at 4°C, and the supernatant was collected and dried on a rotary evaporator (Eppendorf, Hamburg, Germany) at 50°C. The residues were dissolved in 0.5 ml of high-performance liquid chromatography- (HPLC-) grade methanol, collected in tubular glass vials, and air-dried under a chemical hood. An antibiotic assay was performed by dissolving the extracts in methanol to a concentration of 60 mg/ml, and 30 μl of the solution was used for an agar well diffusion assay. The antagonistic activities of the isolates against the *Xcc* and *Xcc-eYFP* strains were determined using the diameter of the inhibition zone. The MIC of streptomycin and ethyl acetate extracts was determined. For minimum bactericidal concentration (MBC) determination, 10 μl of MIC cultures were transferred from the microtiter plates to NA plates and incubated at 28°C for 24 h. The lowest concentrations of the ethyl acetate extract that prevented visible growth of bacteria on NA plates were indicated as the MBCs. Both MIC and MBC were denominated in μg/ml.

### Statistics and Reproducibility

Numeric data are presented as mean ± standard deviation (SD) unless otherwise specified in the figures. Statistical analysis was performed with the software Prism 9. *P*-values and statistical analysis methods are indicated. The correlations of *Xcc-eYFP* and *Xcc* and the correlations of two methods in the bactericide inhibition rates from the plaque neutralization assay were analyzed using a linear regression model, Pearson’s correlation coefficient, and two-tailed *p*-value. One-/two-way ANOVA analyses with the following corresponding multiple comparisons were processed to meet the data analysis requirement with the Tukey’s test [95% confidence interval (CI)] using the software of GraphPad Prism 9 (**p < 0.05, ***p < 0.001, ****p < 0.0001, *nsP*>0.05).

## Results

### Determination of Buffer and Incubation Time

The *Xcc-eYFP* suspension was suspended with ddH_2_O into a final concentration with OD_600_ = 1 (the optical density at 600 nm is equal to 1). The bacterial suspension was pipetted into the black 96-well plate. Then, the optimal excitation/emission wavelength measured by the Spark™ multimode microplate reader (TECAN, Switzerland) has arisen at 485/535 nm. We used four bacterial suspension buffers to reduce the noise signal, including NB, LB, ddH_2_O, and 10 mM MgCl_2_. Those buffers were processed into fluorescence intensity tests using the screened optimal excitation/emission wavelength. The results showed that the autofluorescence intensity of NB is 11,513.67 ± 85.70, and that of LB is 10,632.00 ± 195.85. 10 mM MgCl_2_ and ddH_2_O are 56.67 ± 1.53 and 57.16 ± 1.16, respectively. 10 mM MgCl_2_ and ddH_2_O had the minimum noise signal during detection among the four buffers. Next, *Xcc-eYFP* bacterial suspensions were incubated in different buffer concentrations (OD_600_ = 0.3) for 0, 6, 12, 24, 30, and 36 h (Supplementary Figure 1). The result showed that the fluorescence of *Xcc-eYFP* in the 10 mM MgCl_2_ buffer presented more stable than the other treatments. In addition, we examined the tolerance of *Xcc-eYFP* reporter strains to organic solvents commonly used to dissolve compounds in most drug libraries. We exposed *Xcc-eYFP* suspensions at a concentration of 5 × 10^8^ (CFU/ml) to DMSO, DMF, NB, 10 mM MgCl_2_, and acetone at concentrations ranging from 0 to 100% for 36 h. We found that *Xcc-eYFP* in 10 mM MgCl_2_ and DMSO solvents presented a lower inhibition effect than the other solvents (Supplementary Figure 2). This information is critical to guide decisions regarding the choice of solvents and appropriate concentrations to use in drug screening assays without compromising the vitality of *Xcc-eYFP* reporter strains.

For incubation time determination, we mixed 190 μl *Xcc-eYFP* (suspended in the corresponding solvent as the same as bactericides) with 10 μl of different bactericides in 96-well plates to monitor the bacterial vitality *Xcc-eYFP* dynamically. The plate was incubated at 28°C for a series of time points and then delivered for fluorescent signal detection. The results showed a dynamic fluorescent quenching phenomenon during incubation at different time points. Constant fluorescence intensity of *Xcc-eYFP* was observed at 6 h in all treatments (Supplementary Figure 3). The optimal incubation time for the following experiments is defined as 6 h postinoculation.

### The Correlation Between the Fluorescence Intensity of *Xanthomonas citri* subsp. *citri* Strain and the Alive Bacterial Population

**FIGURE 1:**
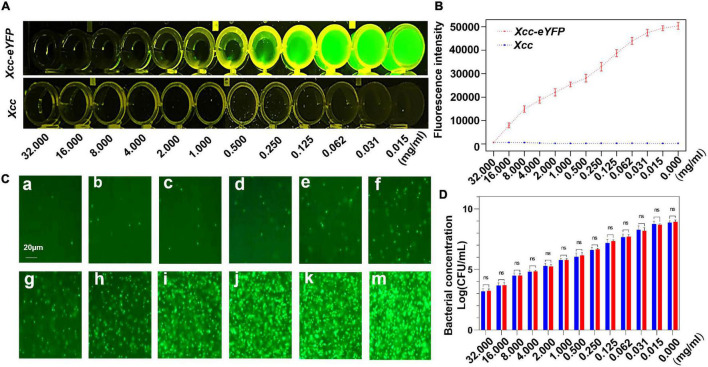
The correlation between fluorescence reading and the alive bacterial population of *Xcc-eYFP*. **(A)** After 6 h of incubation, *Xanthomonas citri* subsp. *citri* (*Xcc*) and *Xcc-eYFP* express fluorescence signals, at 12 concentrations of 30% copper oxychloride (SC), were captured under LUYOR-3145RG irradiation. **(B)** After 6 h of incubation, *Xcc* and *Xcc-eYFP* express fluorescence intensity, at 12 concentrations of 30% copper oxychloride (SC), were captured under the Spark™ multimode microplate reader (TECAN, Switzerland). Means and SDs of five replicates of a representative result are shown. Vertical bars represent the SD of the means. **(C)** *Xcc-eYFP* suspension (OD = 1) was co-incubated with a series of 30% copper oxychloride (SC) concentrations of *a* = 32, *b* = 16, *c* = 8, *d* = 4, *e* = 2, *f* = 1, *g* = 0.5, *h* = 0.25, *i* = 0.125, *j* = 0.062, *k* = 0.031, *m* = 0.015 mg/ml, after 6 h of incubation, The fluorescence of *Xcc-eYFP* footprint was captured through a transmission fluorescence microscope. **(D)** Alive bacterial concentrations of *Xcc* and *Xcc-eYFP* were counted by the plate colony counting method and fluorescent spots counting method. Means and SDs of the three replicates of a representative result are shown. Error bars are representative of the SD from the mean. Statistical analysis was studied with the Tukey’s test (95% confidence interval (CI), *nsP*>0.05). ddH_2_O, double-distilled water.

The counting of colony-forming units based on dilution series of plates on agar media is a common approach to measure bacterial growth from the treatment, but this is time-consuming and laborious. We aimed to simplify and expedite the quantification of *Xcc* by counting fluorescent *Xcc-eYFP* under a fluorescent microscope, and then the correlation between fluorescence intensity and the bacterial population was calculated. To further match the correlation in this study, copper oxychloride was used for evaluation to fit the linear equation. The fluorescence quenching of *Xcc-eYFP* was observed at different concentrations of 30% copper oxychloride (SC). The fluorescence intensity of *Xcc-eYFP* gradually decreased along with increasing bactericide concentration when the wild-type *Xcc* was used as a negative control (Figures 1A,B). The bacterial population of *Xcc-eYFP* stains at different bactericide concentrations was calculated by counting the fluorescent spots under fluorescent microscopy, as previously described (Duan et al., 2021; Figure 1C). The bacterial population of the *Xcc-eYFP* strain calculated by both methods showed high consistency in different groups (Figure 1D). The result showed a typical linear matched relationship among the treatments, changes in the bacterial population of the *Xcc-eYFP* and *Xcc* strains, which calculated the correlation coefficient as 0.998. These data indicate that the fluorescence intensity of *Xcc-eYFP* measured by the Spark™ multimode microplate reader (TECAN, Switzerland) can be used to quantify the bacterial population.

### Comparison of the Conventional Method and the *Xanthomonas citri* subsp. *citri* Strain*-*Based Assay for the Determination of Bactericide Inhibition

**FIGURE 2:**
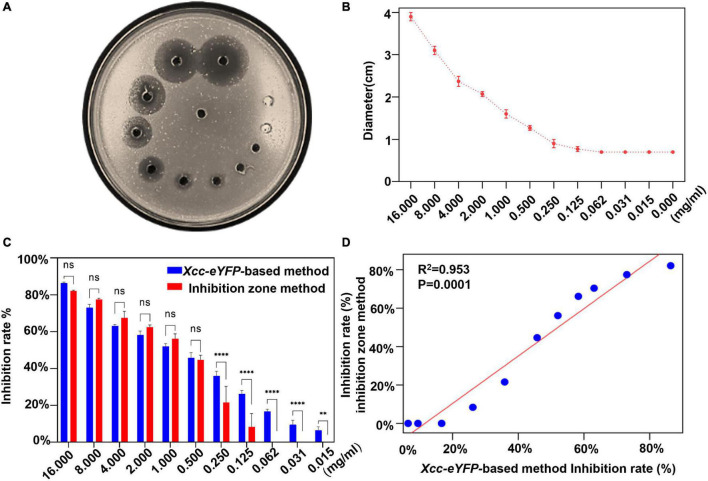
Comparison of bactericidal inhibition rates between reporter-based and conventional inhibition zone methods. **(A)** The image of inhibition zones on an *Xcc-eYFP* plate treated with different 11 concentrations of 30% copper oxychloride (SC) by the inhibition zone method. **(B)** The inhibition zone diameters were caused by 11 concentrations of 30% copper oxychloride (SC) and the control group. Means and SDs of three replicates of a representative result are shown. The vertical bars represent the SD of the means. **(C)** The accuracy comparison between the reporter-based assay and the inhibition zone method to calculate the inhibition rate at a series of 30% copper oxychloride (SC) concentrations. Means and SDs of three replicates of a representative result are shown. Statistical analysis was processed by two-way ANOVA following multiple comparisons with the Tukey’s test (95% CI) using GraphPad Prism 9 (**p < 0.05, ****p < 0.0001, and ns > 0.05).

The inhibition zone method is commonly used to evaluate the antagonism of bactericides against bacteria, but we aimed to simplify and expedite the determination of the bactericide inhibition rate by reading the fluorescence of *Xcc-eYFP*. The inhibition rate of 30% copper oxychloride (SC) was determined by the inhibition zone method (Figure 2A). As the concentration gradually decreased, the diameter of the inhibition zone also gradually decreased (Figure 2B). The inhibition rates calculated by both inhibition zone and reporter-based methods were significantly consistent and met a typical linear matched relationship with a correlation coefficient of 0.953 (Figure 2D). However, it is worth noting that the inhibition threshold value calculated by a reporter-based assay is higher than that calculated by the inhibition zone method (Figure 2C), which indicates that this assay presents more accuracy and can afford the duty of inhibition rate calculation the same as conventional ways.

### Evaluation of the Activity of Different Bactericides

**FIGURE 3:**
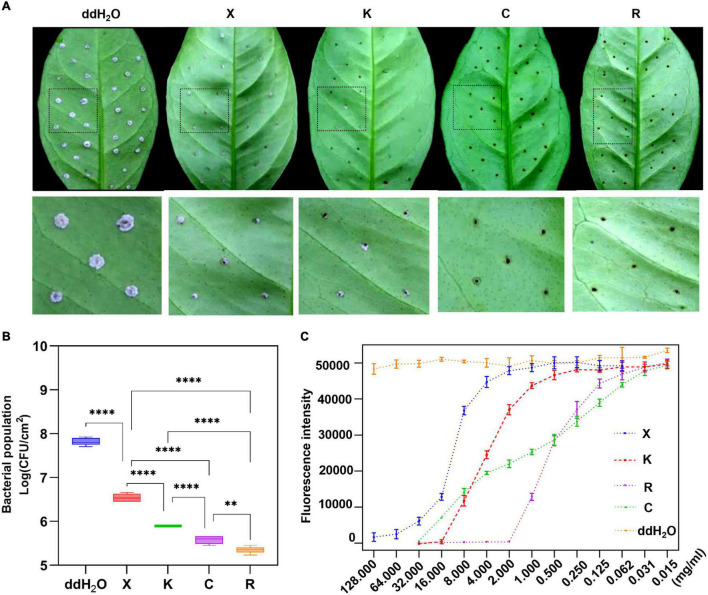
Prevention effect and determination of minimum inhibitory concentrations (MICs) of four individual bactericides with the reporter-based assay. **(A)** After 7 days, the development of citrus bacterial canker (CBC) disease on citrus leaves pretreated with different bactericides was processed in the evaluation of the inhibition rate using the pinprick-inoculation method. **(B)** The result represents the bacterial population among different treatments using the fluorescence spot counting method to calculate the bacterial population of *Xcc-eYFP* at 7 days postinoculation. Experiments were repeated three times with similar results. Error bars are representative of the SD of the mean of five replicates. Statistical analysis was processed by two-way ANOVA following multiple comparisons with the Tukey’s test (95% CI) using GraphPad Prism 9 (**p < 0.05, ****p < 0.0001, *nsP*>0.05). X:1.2% xinjunan acetate (AS); K:33% kasugamycin xine-copper (SC); C:30% copper oxychloride (SC); R: 20% resin acid copper (EW); ddH_2_O: double-distilled water. K:33% kasugamycin xine-copper (SC); X:1.2% xinjunan acetate (AS); C:30% copper oxychloride (SC); R: 20% resin acid copper (EW); ddH_2_O: water. **(C)** After 6 h of incubation, the *Xcc-eYFP* expressing fluorescence intensity in four individual bactericides was captured under the Spark™ Multimode Microplate reader. The *x*-axis represents the concentration of the bactericides. The *y*-axis represents the fluorescence intensity. Means and SDs of nine replicates of a representative result are shown.

The fluorescence intensity of *Xcc-eYFP* suspended in four bactericides at different concentrations was measured. According to the fluorescence curve, it is found that the minimum concentration of 1.2% xinjunan acetate (AS) for complete fluorescence quenching is 128 mg/ml, 33% kasugamycin xine-copper (SC) is 16 mg/ml, 30% copper oxychloride (SC) is 32 mg/ml, and 20% resin acid copper (EW) is 2 mg/ml (Supplementary Figure 4). We used a pinprick-inoculation method to verify CBC disease development on Hamlin sweet orange leaves. The result showed that mock-treated leaves have significant symptoms compared with those of bactericidal treatment (Figure 3A). According to the bacterial growth curve result (Figure 3B), the antimicrobial activity of the four bactericides is ranked as 20% resin acid copper (EW) > 33% kasugamycin xine-copper (SC) > 30% copper oxychloride (SC) > 1.2% xinjunan acetate (AS). The MIC result determined by the conventional method is consistent with the fluorescence intensity change monitored by the Spark™ multimode microplate reader (Supplementary Table 3).

### The Reporter-Based Evaluation of Different Mixture Formulation Bactericides

**FIGURE 4:**
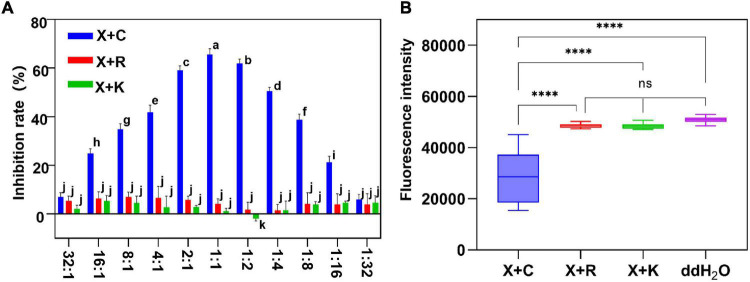
The inhibition evaluation and screening of mixture formulation bactericides. **(A)** The inhibition rate of three different mixture formulations of bactericides was evaluated. The *y*-axis represents the inhibition rate, and the *x*-axis represents the synergistic ratio of bactericides. Statistical analysis was studied by two-way ANOVA following multiple comparisons with the Tukey’s test (95% CI) using GraphPad Prism 9. Different letters in the bars indicate significant differences among the treatments. **(B)** *Xcc-eYFP* expressing fluorescence intensity in three mixture formulations of bactericides was captured under the Spark™ Multimode Microplate reader at 6 h postincubation. The *x*-axis represents three synergetic bactericides, ddH_2_O: double-distilled water, the *y*-axis represents the fluorescence intensity. Statistical analysis was studied by one-way ANOVA following multiple comparisons with the Tukey’s test (95% CI) using GraphPad Prism 9. (****p < 0.0001, *nsP*>0.05). X + C:1:1 mixture formulation of 1.2% xinjunan acetate (AS) and 30% copper oxychloride (SC); X + R:1:1 mixture formulation of 1.2% xinjunan acetate (AS), and 20% resin acid copper (EW). X + K: 1:1 mixture formulation of 1.2% xinjunan acetate (AS) and 33% kasugamycin xine-copper (SC).

To screen the optimal ratio of cocktail combination bactericides, we mixed the formulations of 1.2% xinjunan acetate (AC) (0.5 mg/ml) with three copper bactericides of 33% kasugamycin xine-copper (SC) (0.03 mg/ml), 20% resin acid copper (EW) (0.03 mg/ml), and 20% resin acid copper (EW) (0.25 mg/ml). The dynamic fluorescence intensity of *Xcc-eYFP* co-incubated with different bactericides was monitored (Figure 4A). With a gradual increase in the volume ratios of three mixture formulation bactericides, the fluorescence intensity in the combination of 1.2% xinjunan acetate (AC) (0.5 mg/ml) and 30% copper oxychloride (SC) (0.03 mg/ml) changed obviously and in the other two compounds was relatively stable. According to the computational formula of inhibition rate, the synergistic effect of 1.2% xinjunan acetate (AC) (0.5 mg/ml) and 30% copper oxychloride (SC) (0.03 mg/ml) reaches the peak when the volume ratio was 1:1. It is worth noting that when 20% resin acid copper (EW) (0.03 mg/ml), 33% kasugamycin xine-copper (SC) (0.25 mg/ml) and 1.2% xinjunan acetate (AC) (0.5 mg/ml) are compounded, the synergistic effect does not become robust as the volume ratio varies (Figure 4B). When the volume ratio of 1.2% xinjunan acetate (AC) (0.5 mg/ml) and 30% copper oxychloride (SC) (0.03 mg/ml) is 1:1, the inhibition rate of the compound bactericide is 67.75% overtop the 1.50 and 1.24% of the two individual bactericides (Supplementary Figure 4A). Therefore, the amount of copper-based bactericides used can be reduced to 55.8% under the same inhibition level. In addition, the alive *Xcc-eYFP* and *Xcc* population co-incubated with 11 synergistic rates was calculated. With the different synergistic proportion of 1.2% xinjunan acetate (AC) (0.5 mg/ml) and 30% copper oxychloride (SC) (0.03 mg/ml), the inhibition rates and alive *Xcc-eYFP* and *Xcc* strain population were dramatically altered, but with totally opposite change trend (Supplementary Figure 4B). The inhibition rate calculated here is consistent with the MIC determination that the results showed that the 1:1 synergistic bactericide of 2% xinjunan acetate (AC) (0.5 mg/ml) and 30% copper oxychloride (SC) (0.03 mg/ml) had the lowest MICs of 1 mg/ml for *Xcc* and *Xcc-eYFP* strains (Supplementary Table 3).

**FIGURE 5:**
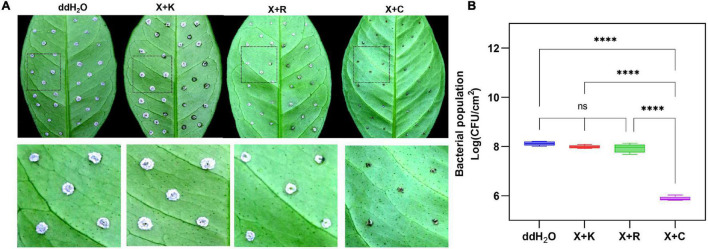
Indoor prevention effect of different mixture combinations of bactericides. **(A)** After 7 days, CBC development on citrus leaves was pretreated with three different 1:1 synergistic bactericides using the pinprick-inoculation method. The experiments were repeated three times with similar results. **(B)** The result represents the bacterial population among the different treatments using the fluorescence spot counting method at 7 days postinoculation. The bar value shown is the mean of three independent experiments. The vertical bars represent the SD of the means. Statistical analysis was studied by one-way ANOVA following multiple comparisons with the Tukey’s test (95% CI) using GraphPad Prism 9 (****p < 0.0001, *nsP*>0.05). X + C:1:1 mixture formulation of 1.2% xinjunan acetate (AS) and 30% copper oxychloride (SC); X + R:1:1 mixture formulation of 1.2% xinjunan acetate (AS), and 20% resin acid copper (EW). X + K: 1:1 mixture formulation of 1.2% xinjunan acetate (AS) and 33% kasugamycin xine-copper (SC); ddH_2_O: double-distilled water.

**FIGURE 6:**
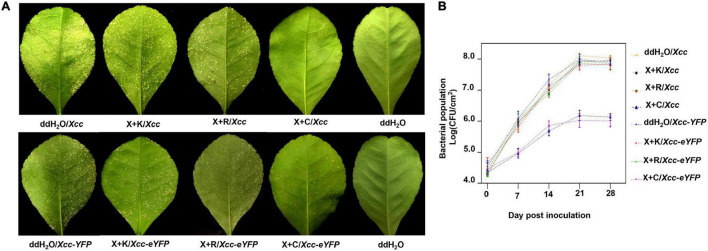
Outdoor prevention effect of the mixture formulation of bactericides against CBC. **(A)** Images represent the development of citrus canker symptoms spray-inoculated by *Xcc* and *Xcc-eYFP* [about 5 × 10^8^ colony-forming units/ml (CFU/ml)] and the corresponding bactericides 28 days postinoculation. **(B)** The bacterial growth curve of *Xcc* and *Xcc-eYFP* strain populations on Lemon leaves above. *Xc*c and *Xcc-eYFP* bacterial cells were extracted from the leaves at different time points after inoculation and quantified using the bacterial counting plate method and bacterial fluorescence spot counting method. The values shown are the means of five repeats and SDs. All assays were repeated three times with similar results. X + C: 1:1 mixture formulation of 1.2% xinjunan acetate (AS) and 30% copper oxychloride (SC); X + K: 1:1 mixture formulation of 1.2% xinjunan acetate (AS) and 33% kasugamycin xine-copper (SC); X + R:1:1 mixture formulation of 1.2% xinjunan acetate (AS) and 20% resin acid copper (EW); ddH_2_O: double-distilled water.

The pinprick-inoculation method was used to evaluate the indoor protective effect of bactericides (Figure 5A). The results showed that the synergistic bactericide of 1.2% xinjunan acetate (AC) (0.5 mg/ml) and 30% copper oxychloride (SC) (0.03 mg/ml) at 1:1 has significant protective effects of inhibiting the bacterial growth of *Xcc* compared with those of the other two treatments and mock-treated (Figure 5B). The result of the outdoor protective effect showed that the 1:1 combination of 1.2% xinjunan acetate (AC) (0.5 mg/ml) and 30% copper oxychloride (SC) (0.25 mg/ml) was able to reduce the development of canker symptoms on lemon leaves, as evidenced by the decrease in the number of lesions compared with the positive control (Figure 6A). Treatment of the lemon leaves with a 1:1 combination of 1.2% xinjunan acetate (AC) (0.5 mg/ml) and 30% copper oxychloride (SC) (0.25 mg/ml) resulted in an approximate 2.0 log reduction in the bacterial population compared with the untreated control. However, the treatment of lemon leaves with two 1:1 combination of 1.2% xinjunan acetate (AC) (0.5 mg/ml) compound 33% kasugamycin xine-copper (SC) (0.25 mg/ml), and 20% resin acid copper (EW) (0.03 mg/ml) did not reduce in the number of CFU compared with the untreated control (Figure 6B).

### Screening and Evaluation of Antagonistic Bacterial Strains Against *Xanthomonas citri* subsp. *citri*

**FIGURE 7:**
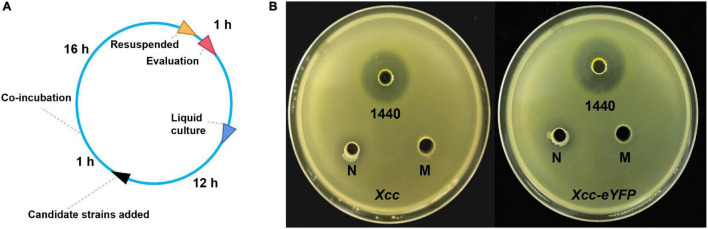
The screening and evaluation process of *Xcc*-antagonistic bacterial strain. **(A)** Timelines of screening and evaluation processes of *Xcc*-antagonistic bacterial strains. **(B)** The antibacterial activity of the extraction was measured using the agar well diffusion assay. M, methanol control; 1440, ethyl acetate extract of antagonistic bacteria 1440. N: Negative control, ethyl acetate extract of *Agrobacterium* strain EHA105. All assays were repeated three times with similar results.

**FIGURE 8:**
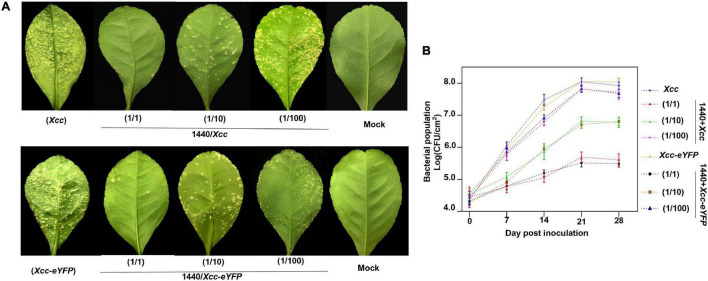
Evaluation of outdoor protective effect of *Burkholderia* strain-1440 against CBC. **(A)** CBC symptom development on lemon leaves co-sprayed with *Xcc* and *Xcc-eYFP* and *Burkholderia* strain-1440. Images are representative of four independent replicates at 28 days postinoculation. **(B)** The bacterial growth curve of *Xcc* and *Xcc-eYFP* on the abovementioned leaves using the bacterial counting plate method and fluorescence spot counting method. The values shown are the means of five repeats and SDs. All assays were repeated three times with similar results. *Xcc*: strain *Xcc* alone; 1440/*Xcc*: (1/1): *Burkholderia* strain-1440 inoculated simultaneously with *Xcc* at the ratio of 1–1; (1/10): *Burkholderia* strain-1440 spray-inoculated simultaneously with *Xcc* at the ratio of 1–10; (1/100): *Burkholderia* strain-1440 spray-inoculated simultaneously with *Xcc* at the ratio of 1–100. *Xcc-eYFP*: *Xcc-eYFP* spray-inoculated alone; 1440/*Xcc-eYFP*: (1/1): *Burkholderia* strain-1440 inoculated simultaneously with *Xcc-eYFP* at the ratio of 1–1; (1/10): *Burkholderia* strain-1440 inoculated simultaneously with *Xcc-eYFP* at the ratio of 1–10; (1/100): *Burkholderia* strain-1440 inoculated simultaneously with *Xcc-eYFP* at the ratio of 1–100; Mock: ddH_2_O.

Microbes can secrete antibacterial components to restrain bacterial growth. Here, we establish a timeline and processes for *Xcc*-antagonistic bacteria and agents screening and evaluation (Figure 7A). In this study, an *Xcc*-antagonistic *Burkholderia* bacterial strain-1440 (Hereafter named *Burkholderia* strain-1440) was screened and applied for antagonism evaluation. *Burkholderia* strain-1440 (GenBank: OM943749) affiliated with *Burkholderia stabilis*, based on the result of 16S rDNA analysis, showed high inhibition against *Xcc*. In the conventional inhibition zone method, the *Burkholderia* strain-1440 derivative, secondary metabolites have high performance against *Xcc*/*Xcc-eYFP* (Figure 7B). Ethyl acetate extraction of *Burkholderia* strain-1440 (24 h at 28°C in the LB medium) exhibited stable thermostability (incubated at 60°C for 4 h) and inhibition activity, with an inhibition zone ranging from 22.49 ± 1.36 to 24.35 ± 1.36 mm. The extraction of *Burkholderia* strain-1440 was processed for metabolomics analysis by the UPLC-MS/MS. The result reveals that several potential antibacterial secondary metabolites were hatched from the extract of *Burkholderia* strain-1440 (Supplementary Data Sheet 1). The results of the outdoor protective effect showed that *Burkholderia* strain-1440 significantly inhibits the CBC symptom development and bacterial growth obviously (Figures 8A,B). Spraying treatment of leaves with a 1:1 combination of *Burkholderia* strain-1440 and *Xcc/Xcc-eYFP* strain resulted in an approximate 2.0 log reduction in the number of CFU compared with the untreated control. Treatment with a 1:10 combination resulted in an approximately 1.0 log reduction in the number of CFU/square centimeter, while treatment with a 1:100 combination resulted in no reduction compared with the untreated control postinoculation. The inhibition result reveals that *Burkholderia* strain-1440 strain and secondary derived metabolites can be applied for CBC control and management *via* the protective and therapeutic application.

## Discussion

Fluorescent reporters have been applied to evaluate the antimicrobial activity of antimicrobial components against pathogenic microorganisms (Cooksey et al., 1995; Arain et al., 1996; Shawar et al., 1997; Gupta et al., 2017) to meet the high-throughput screening purpose, but surprisingly for *Xcc*. Even though the screening of antibacterial components for *Xanthomonas* has been reported (Jiang et al., 2020; Le et al., 2021), methods applied in those studies are conventional ways, such as antimicrobial spectrum experiments or the inhibition zone method. The need for new antimicrobial components to reduce the anxious issues of copper-resistant and environment contamination requires robust methods for screening and evaluation. So we developed a rapid evaluation method for antimicrobial components screening and evaluation to *Xcc* by using a fluorescent protein-labeled *Xcc-eYFP*, which can footprint the vitality of *Xcc* during antibacterial component screening with the Spark™ multimode microplate reader. Notably, a previous study (Duan et al., 2021) proved that the eYFP protein does not affect the growth and virulence of *Xcc*. The MIC calculation results in the conventional method are notably equated to the corresponding base value in a reporter-based assay. It is more robust than the conventional inhibition zone and colony counting method *via* eliminating the need for plating on agar media, reducing labor and time-cost. Meanwhile, this method highly showed accuracy during inhibition evaluation to meet the low threshold of bactericide inhibition rate using the Spark™ multimode microplate reader (TECAN, Switzerland).

In this study, one alkyl polyamine-type bactericide: 33% kasugamycin xine-copper (SC), and three copper-based bactericides: 1.2% xinjunan acetate (AS), C:30% copper oxychloride (SC), and 20% resin acid copper (EW) were processed in the construction and evaluation of the method. We calculated the inhibition activity of bactericides and ranked them in the following order: 20% resin acid copper (EW) > 33% kasugamycin xine-copper (SC) > 30% copper oxychloride (SC) > 1.2% xinjunan acetate (AS). The test of preventive effect by the outdoor spray method is consistent with the evaluation results. On the other hand, based on the evaluation of the combination with a different ratio, we screened out a well-performance combination ratio 1:1 combination of 1.2% xinjunan acetate (AC) (0.5 mg/ml) and 30% copper oxychloride (SC) (0.25 mg/ml). The combination bactericide can reduce up to 55.8% of copper component application dosage with the same protective effect as an individual bactericide, reducing the development of canker symptoms compared with the positive control. Usually, partial cocktail bactericide combinations present better performance in the filed application than the signal agent, but the ratio based on the experientialism cannot reach the optimal synergistic effect. The optimal ratio of bactericides formulation will enhance the prevention effect. Meanwhile, the application of *Xcc*-antagonistic bacterial strain and derived metabolites will significantly reduce the application dosage of copper-based bactericides, further relieving the anxiety problems of copper-resistant and environmental contamination. Our study will accelerate the screening and evaluation process of new antibacterial ingredients to meet the requirements of CBC control and management.

However, some tips need more attention during screening and evaluation. Firstly, partial solvents/buffers/bactericides generate an autofluorescence that will disturb the honest signal value reading by the spark multimode microplate reader. In this case, we suggest increasing the dilution, setting up multiple controls, or changing the solvent. Secondly, the inhibition rate evaluated from the indoor inhibition rate test is appropriately different from the result calculated from the field application because factors such as the field climate, the attachment, and penetration capacity of the drug may be different from the actual application of antibacterial products in the field. However, whether the antibacterial compound has an anticipated preventive effect in the field, the indoor inhibition rate results obtained by the *Xcc-eYFP* assay can provide essential reference data for field medication. Some components remained valid hits with inhibition above the 50% threshold in the reporter-based assay, but failed in the oxford-cup inhibition zone method. Despite the apparent inhibition of *Xcc* growth by the luminescence readout could be the result of different growth conditions or bacteriostatic mode that would dampen the increase in luminescence but not the decrease in the inhibition zone method. Some candidates do not appear rapid bactericidal activity as evident at low inhibition by the reporter-based assay, which could be due to a long duration of the fluorescent reporter signal post-treatment.

In summary, we have developed a new method to evaluate and screen antibacterial components against *Xcc*. This method has been successfully used to rank the inhibition rates of commercial bactericides, combination bactericides and *Xcc*-antagonistic bacterial strains. A well-performance *Xcc*-prevented combination of bactericide and a antagonistic bacterial strain was screened. This method can be applied for, in particular, a significant number of candidates, screening and evaluation of new/combination bactericides. The application dosage of copper-based bactericides can be reduced by new/combination bactericides, which will prevent the occurrence of ecosystem contamination and copper-resistant bacterial strains during the control and management of CBC.

## Data Availability Statement

The original contributions presented in the study are included in the article/Supplementary Material, further inquiries can be directed to the corresponding author/s.

## Author Contributions

SD contributed to the conception and design of the study and wrote the first draft of the manuscript. YL, ZX, RL, and SC organized the database. MB and ML performed the statistical analysis. LL kept plant maintenance. YL and HM wrote sections of the manuscript. All authors contributed to manuscript revision, read, and approved the submitted version.

## Conflict of Interest

The authors declare that the research was conducted in the absence of any commercial or financial relationships that could be construed as a potential conflict of interest.

## Publisher’s Note

All claims expressed in this article are solely those of the authors and do not necessarily represent those of their affiliated organizations, or those of the publisher, the editors and the reviewers. Any product that may be evaluated in this article, or claim that may be made by its manufacturer, is not guaranteed or endorsed by the publisher.
