# Supplementary material for: A Fluorescent Reporter-Based Evaluation Assay for Antibacterial Components Against Xanthomonas citri subsp. citri
Source: Front Microbiol. 2022 May 4;13:864963. doi: 10.3389/fmicb.2022.864963 (PMC9114712; doi:10.3389/fmicb.2022.864963)
Supplement: Supplementary file 2 [file Presentation_1.PPTX]

## Slide 1
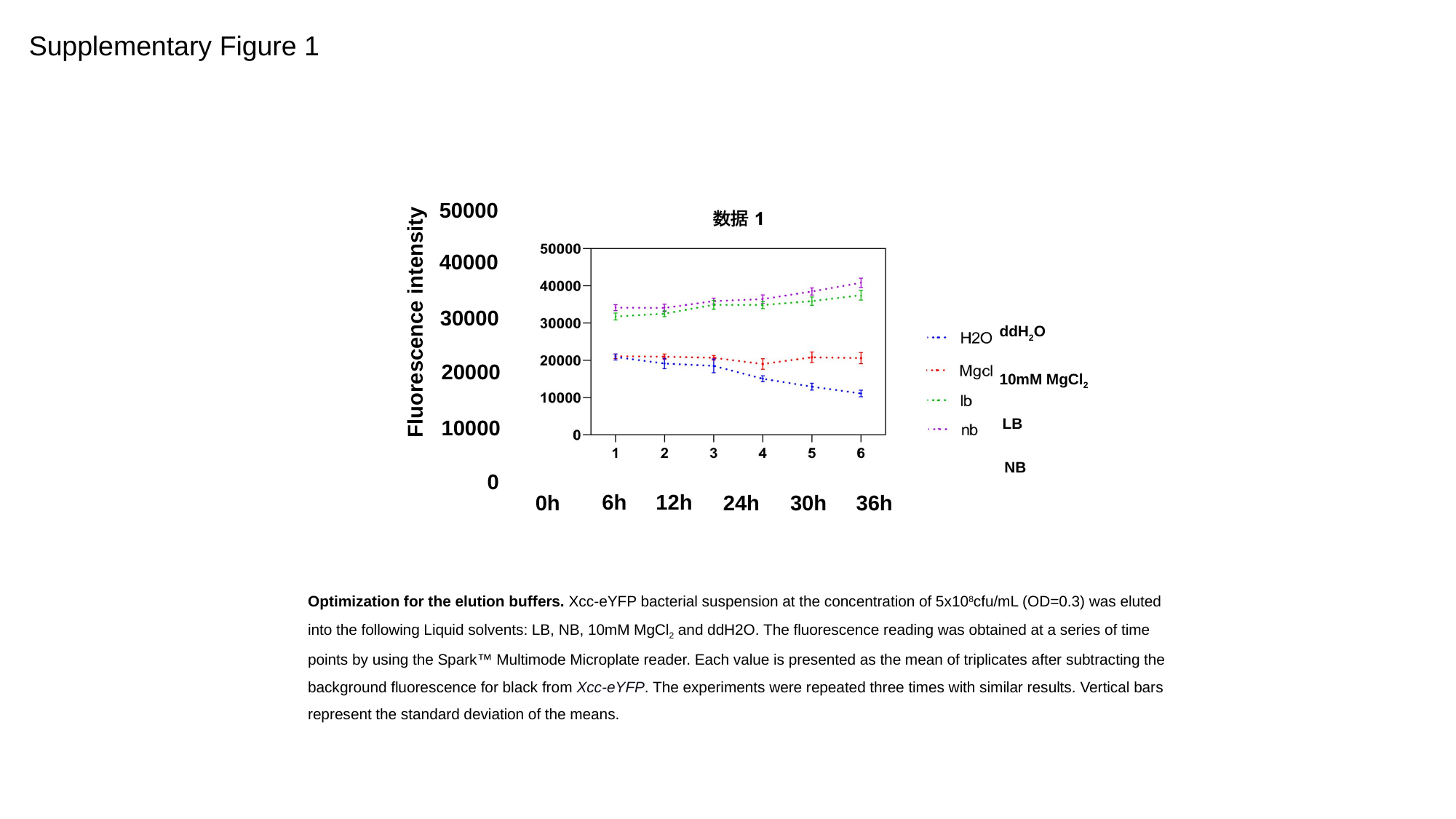

Supplementary Figure 1
50000
40000
 Fluorescence intensity
30000
ddH2O
20000
10mM MgCl2
LB
10000
NB
0
12h
6h
24h
36h
30h
0h
Optimization for the elution buffers. Xcc-eYFP bacterial suspension at the concentration of 5x10^8^cfu/mL (OD=0.3) was eluted into the following Liquid solvents: LB, NB, 10mM MgCl2 and ddH2O. The fluorescence reading was obtained at a series of time points by using the Spark™ Multimode Microplate reader. Each value is presented as the mean of triplicates after subtracting the background fluorescence for black from Xcc-eYFP. The experiments were repeated three times with similar results. Vertical bars represent the standard deviation of the means.

## Slide 2
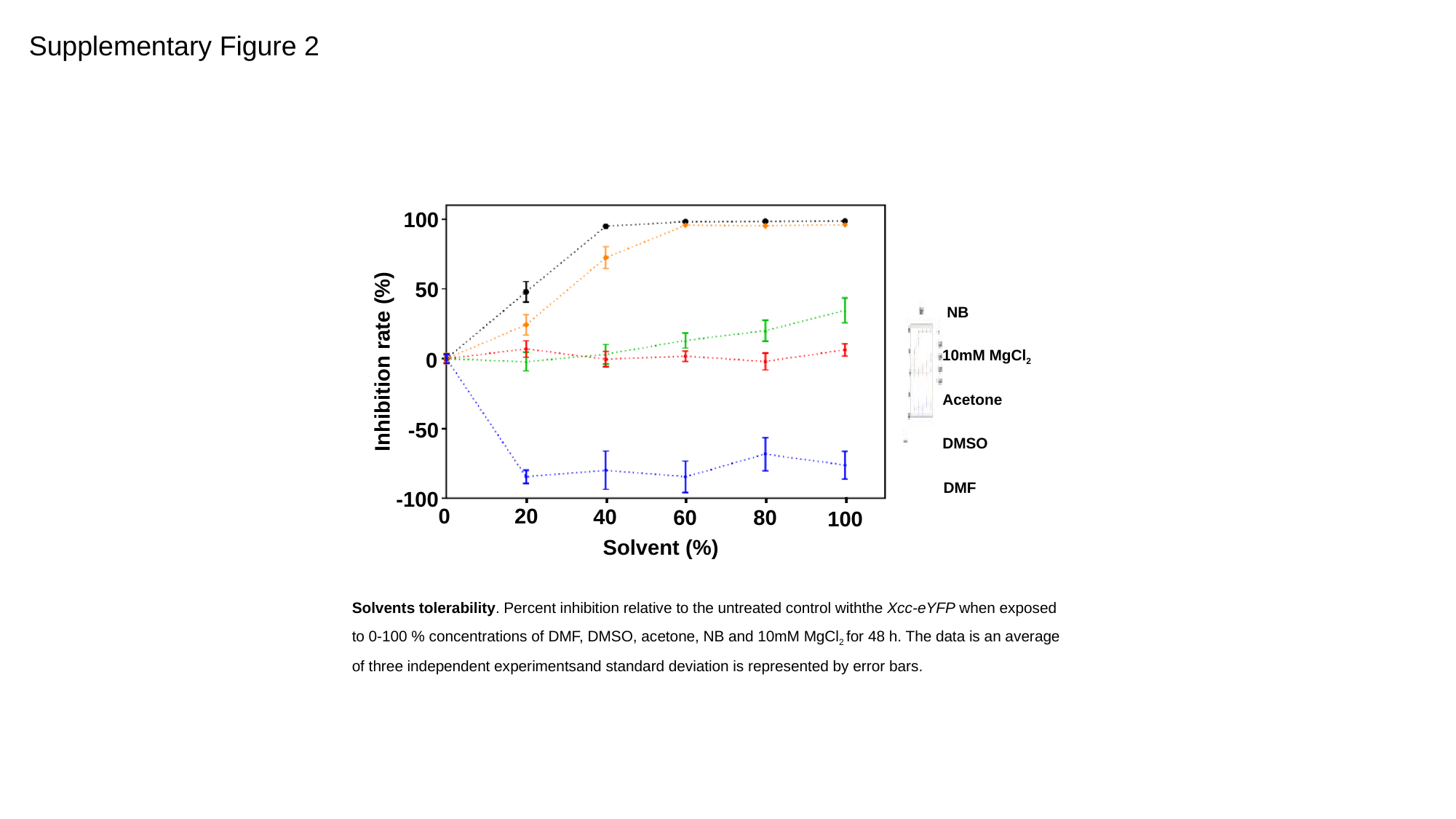

Supplementary Figure 2
100
50
NB
Inhibition rate (%)
10mM MgCl2
0
Acetone
-50
DMSO
DMF
-100
20
0
40
60
80
100
Solvent (%)
Solvents tolerability. Percent inhibition relative to the untreated control withthe Xcc-eYFP when exposed to 0-100 % concentrations of DMF, DMSO, acetone, NB and 10mM MgCl2 for 48 h. The data is an average of three independent experimentsand standard deviation is represented by error bars.

## Slide 3
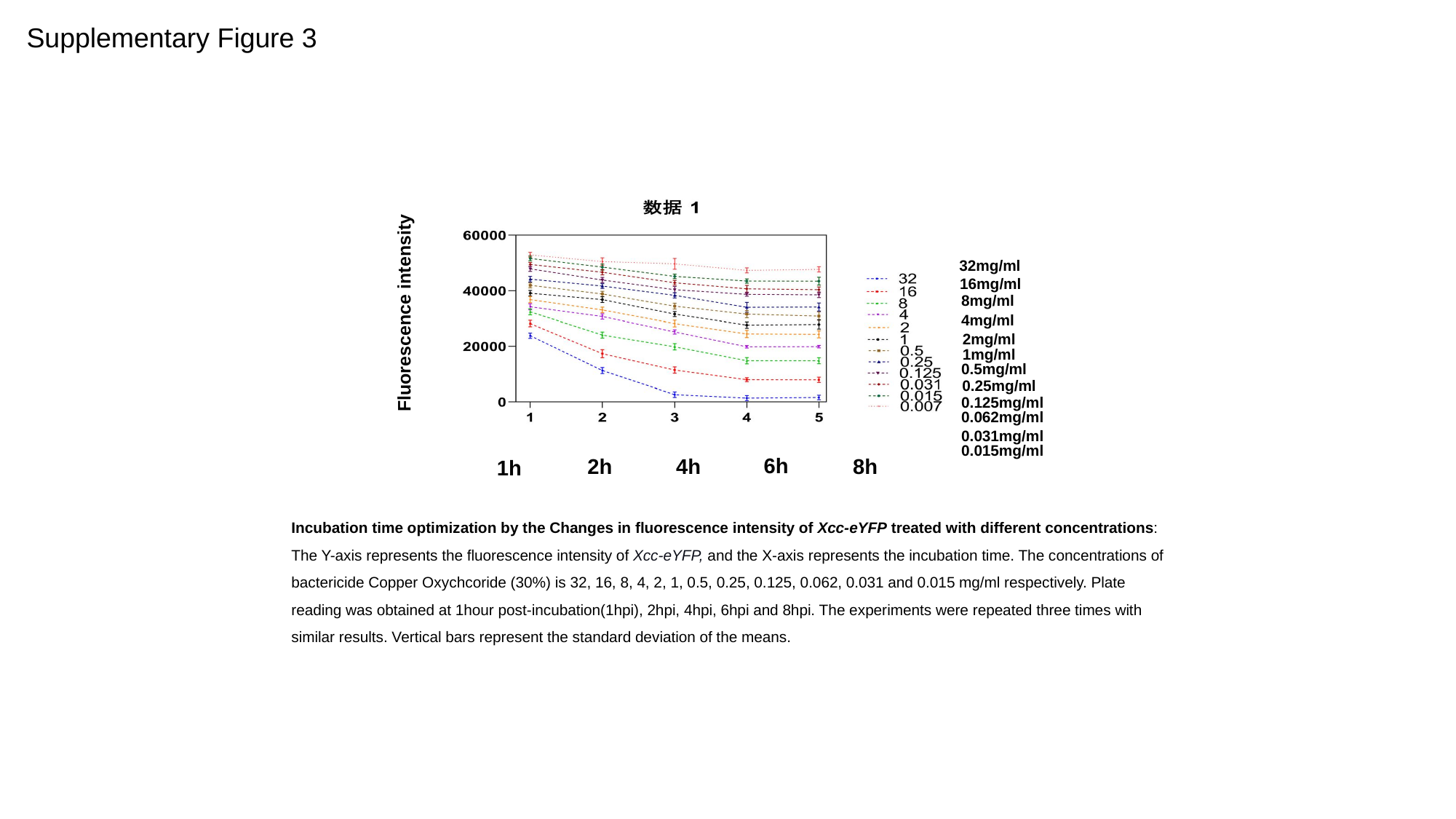

Supplementary Figure 3
32mg/ml
16mg/ml
 Fluorescence intensity
8mg/ml
4mg/ml
2mg/ml
1mg/ml
0.5mg/ml
0.25mg/ml
0.125mg/ml
0.062mg/ml
0.031mg/ml
0.015mg/ml
6h
8h
4h
2h
1h
Incubation time optimization by the Changes in fluorescence intensity of Xcc-eYFP treated with different concentrations: The Y-axis represents the fluorescence intensity of Xcc-eYFP, and the X-axis represents the incubation time. The concentrations of bactericide Copper Oxychcoride (30%) is 32, 16, 8, 4, 2, 1, 0.5, 0.25, 0.125, 0.062, 0.031 and 0.015 mg/ml respectively. Plate reading was obtained at 1hour post-incubation(1hpi), 2hpi, 4hpi, 6hpi and 8hpi. The experiments were repeated three times with similar results. Vertical bars represent the standard deviation of the means.

## Slide 4
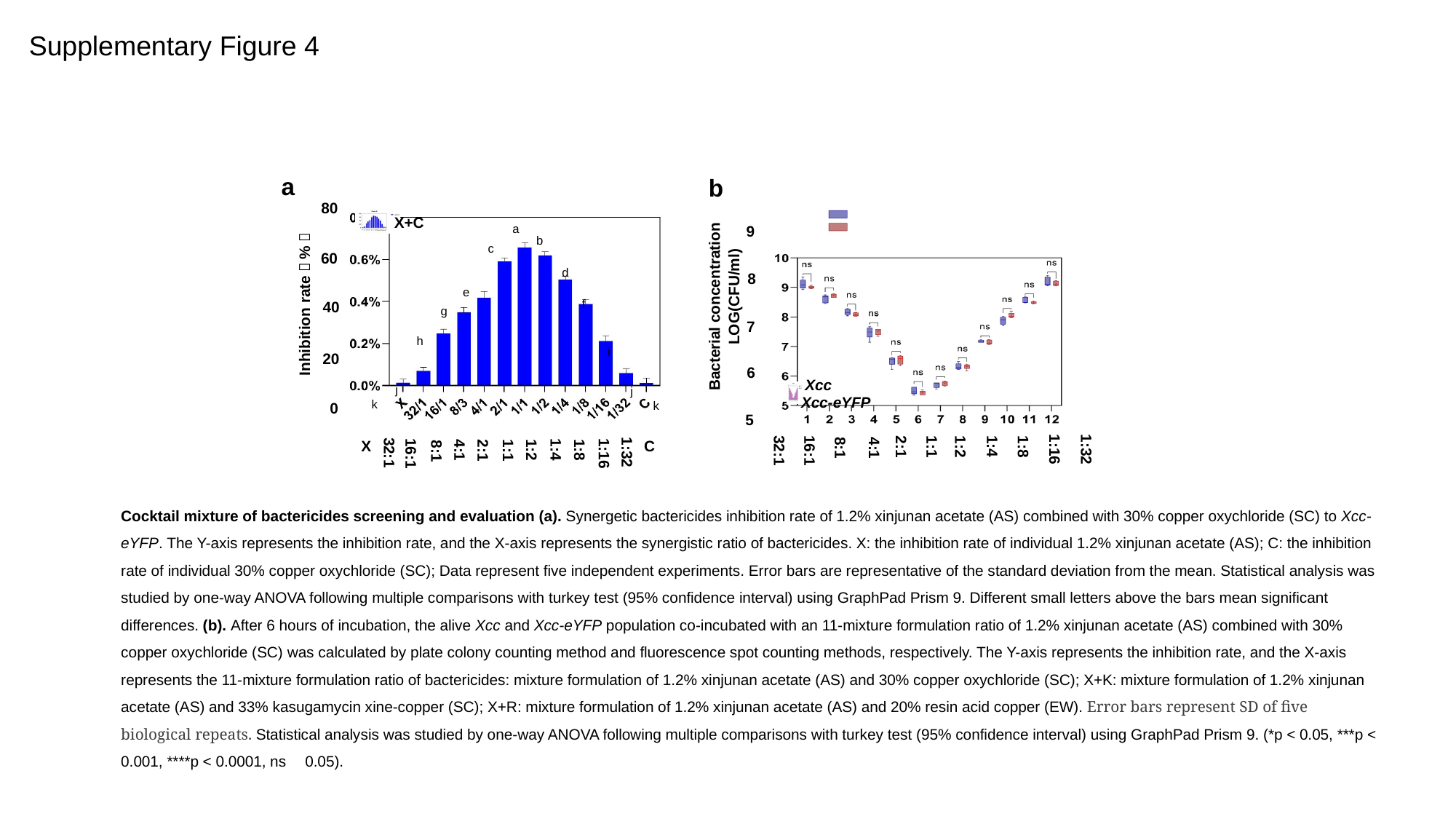

Supplementary Figure 4
a
b
80
X+C
60
Inhibition rate（%）
40
20
0
X
C
1:4
1:32
1:2
4:1
2:1
1:1
8:1
1:8
1:16
32:1
16:1
9
8
LOG(CFU/ml)
Bacterial concentration
7
6
Xcc
Xcc-eYFP
5
1:4
2:1
1:1
1:2
1:32
8:1
4:1
1:8
1:16
32:1
16:1
a
b
c
d
e
f
g
h
i
j
j
k
k
Cocktail mixture of bactericides screening and evaluation (a). Synergetic bactericides inhibition rate of 1.2% xinjunan acetate (AS) combined with 30% copper oxychloride (SC) to Xcc-eYFP. The Y-axis represents the inhibition rate, and the X-axis represents the synergistic ratio of bactericides. X: the inhibition rate of individual 1.2% xinjunan acetate (AS); C: the inhibition rate of individual 30% copper oxychloride (SC); Data represent five independent experiments. Error bars are representative of the standard deviation from the mean. Statistical analysis was studied by one-way ANOVA following multiple comparisons with turkey test (95% confidence interval) using GraphPad Prism 9. Different small letters above the bars mean significant differences. (b). After 6 hours of incubation, the alive Xcc and Xcc-eYFP population co-incubated with an 11-mixture formulation ratio of 1.2% xinjunan acetate (AS) combined with 30% copper oxychloride (SC) was calculated by plate colony counting method and fluorescence spot counting methods, respectively. The Y-axis represents the inhibition rate, and the X-axis represents the 11-mixture formulation ratio of bactericides: mixture formulation of 1.2% xinjunan acetate (AS) and 30% copper oxychloride (SC); X+K: mixture formulation of 1.2% xinjunan acetate (AS) and 33% kasugamycin xine-copper (SC); X+R: mixture formulation of 1.2% xinjunan acetate (AS) and 20% resin acid copper (EW). Error bars represent SD of five biological repeats. Statistical analysis was studied by one-way ANOVA following multiple comparisons with turkey test (95% confidence interval) using GraphPad Prism 9. (*p < 0.05, ***p < 0.001, ****p < 0.0001, ns＞0.05).

## Slide 5
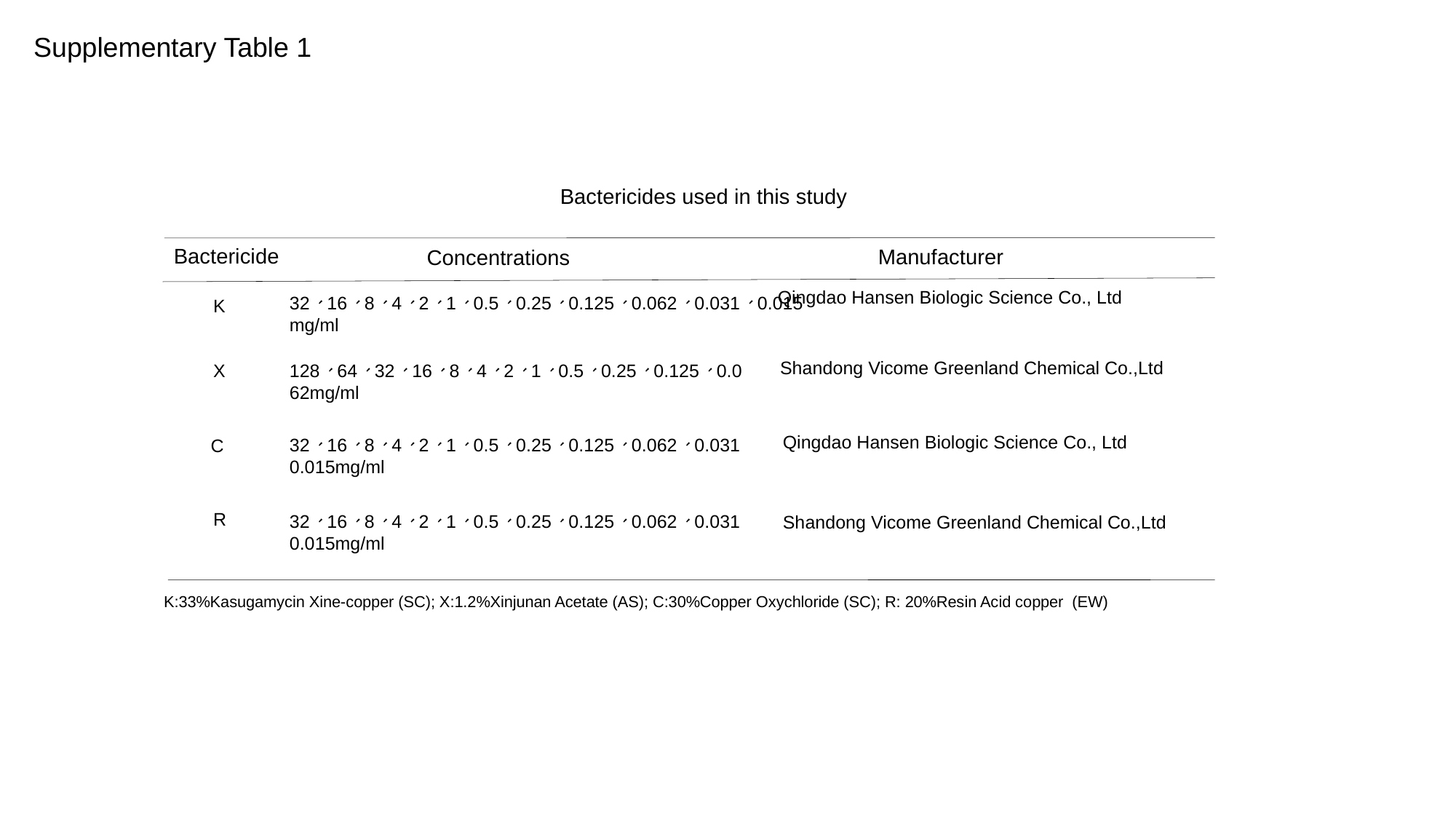

Supplementary Table 1
Bactericides used in this study
Bactericide
Manufacturer
Concentrations
Qingdao Hansen Biologic Science Co., Ltd
32、16、8、4、2、1、0.5、0.25、0.125、0.062、0.031、0.015mg/ml
K
X
128、64、32、16、8、4、2、1、0.5、0.25、0.125、0.062mg/ml
32、16、8、4、2、1、0.5、0.25、0.125、0.062、0.031 0.015mg/ml
Qingdao Hansen Biologic Science Co., Ltd
C
R
32、16、8、4、2、1、0.5、0.25、0.125、0.062、0.031 0.015mg/ml
K:33%Kasugamycin Xine-copper (SC); X:1.2%Xinjunan Acetate (AS); C:30%Copper Oxychloride (SC); R: 20%Resin Acid copper (EW)
Shandong Vicome Greenland Chemical Co.,Ltd
Shandong Vicome Greenland Chemical Co.,Ltd

## Slide 6
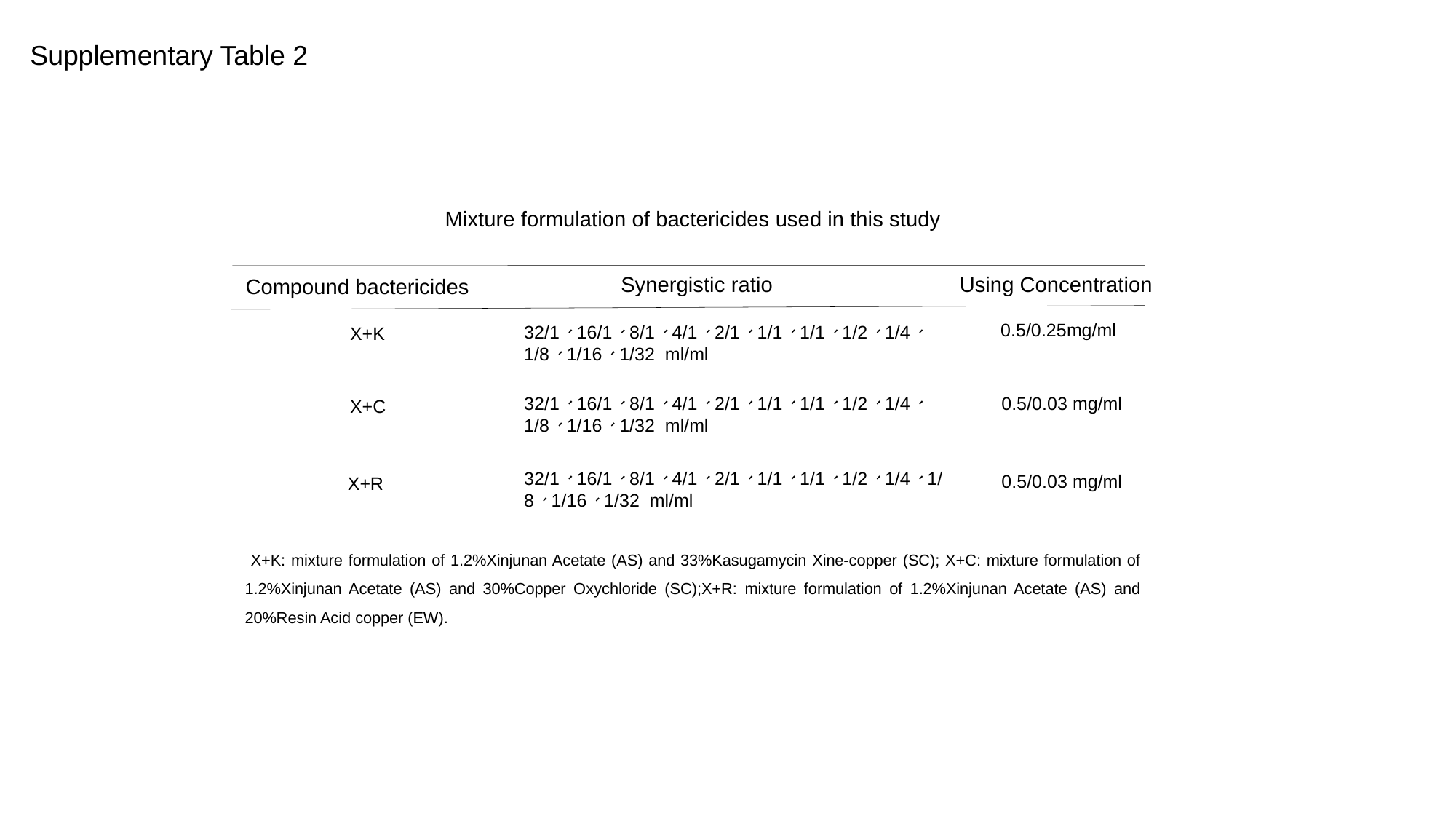

Supplementary Table 2
Mixture formulation of bactericides used in this study
32/1、16/1、8/1、4/1、2/1、1/1、1/1、1/2、1/4、1/8、1/16、1/32 ml/ml
X+K
X+C
X+R
Synergistic ratio
Using Concentration
Compound bactericides
0.5/0.25mg/ml
32/1、16/1、8/1、4/1、2/1、1/1、1/1、1/2、1/4、1/8、1/16、1/32 ml/ml
0.5/0.03 mg/ml
32/1、16/1、8/1、4/1、2/1、1/1、1/1、1/2、1/4、1/8、1/16、1/32 ml/ml
0.5/0.03 mg/ml
 X+K: mixture formulation of 1.2%Xinjunan Acetate (AS) and 33%Kasugamycin Xine-copper (SC); X+C: mixture formulation of 1.2%Xinjunan Acetate (AS) and 30%Copper Oxychloride (SC);X+R: mixture formulation of 1.2%Xinjunan Acetate (AS) and 20%Resin Acid copper (EW).

## Slide 7
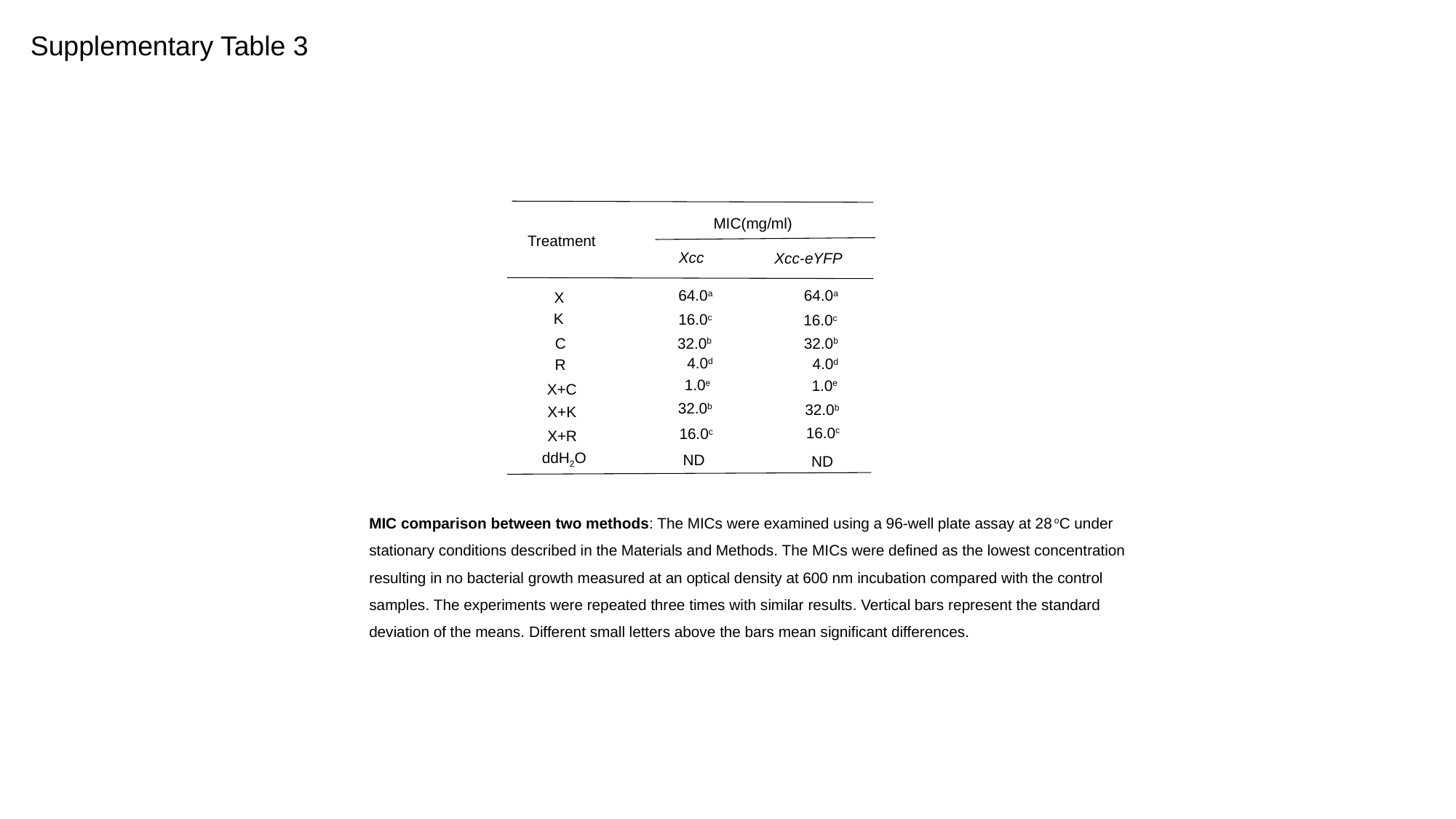

Supplementary Table 3
MIC(mg/ml)
Treatment
Xcc
Xcc-eYFP
64.0a
16.0c
32.0b
4.0d
1.0e
32.0b
16.0c
64.0a
16.0c
32.0b
4.0d
1.0e
32.0b
16.0c
X
K
C
R
X+C
X+K
X+R
ddH2O
ND
ND
MIC comparison between two methods: The MICs were examined using a 96-well plate assay at 28 oC under stationary conditions described in the Materials and Methods. The MICs were defined as the lowest concentration resulting in no bacterial growth measured at an optical density at 600 nm incubation compared with the control samples. The experiments were repeated three times with similar results. Vertical bars represent the standard deviation of the means. Different small letters above the bars mean significant differences.
